# The novel IMAGE001 genotyping array as a valuable alternative for genetic diversity screening in chicken: a demonstration in a local chicken breed in Belgium

**DOI:** 10.1016/j.psj.2023.103221

**Published:** 2023-10-21

**Authors:** Roel Meyermans, Olivier Heylen, Jessica Bouhuijzen Wenger, Jan Martens, Nadine Buys, Steven Janssens

**Affiliations:** ⁎ Department of Biosystems, Center for Animal Breeding and Genetics, KU Leuven, 3001 Leuven, Belgium; † OTEAS Consulting & Statistics (Agriculture, Environment & Ecology), 2221 Booischot, Belgium; ‡ Steunpunt Levend Erfgoed vzw, 9860 Oosterzele, Belgium

**Keywords:** genetic diversity, single nucleotide polymorphism, inbreeding, run of homozygosity, Turkey-headed Maline

## Abstract

Screening for genetic diversity in livestock species breeds is of utmost importance, especially for local, small populations that are at the risk of extinction. Luckily, recent developments in technology increase access to genotyping, also for numerically small breeds. One of these new technologies is the IMAGE001 single nucleotide polymorphism genotyping array that includes markers for 6 different species (cow, pig, sheep, chicken, horse and goat). For our current study, we studied the Turkey-headed Malines chicken, a local chicken breed in Belgium, for the first time. A total of 110 animals were genotyped, together with 29 samples from 4 supposedly related breeds. The genotypes were used to assess the genetic diversity of this local breed. Our analysis revealed an average inbreeding coefficient of 0.20 through runs of homozygosity analysis, and effective population size estimation based on linkage disequilibrium indicated a low genetic diversity (*N*_e_ = 34). Moreover, a principal component analysis and a genetic differentiation study (*F*_ST_) were performed using these marker data to position the Turkey-headed Malines relative to the 4 other indigenous Belgian chicken breeds. Finally, we discussed the practical implications of the overlap between the IMAGE001 array and other existing chicken genotyping arrays. This study is the first use of the novel IMAGE001 array to evaluate a local chicken breed, and demonstrates it as a viable option for genomic characterization a breed. Moreover, with this research, we are able to provide a good basis for further evaluation of the Belgian chicken heritage.

## INTRODUCTION

The preservation of genetic diversity in livestock species breeds is vital, particularly for small and local populations that face the threat of extinction (Meuwisen and Oldenbroek, 2017). Local chicken breeds, like many other livestock breeds, are frequently endangered (FAO, 2013). The Food and Agriculture Organization of the United Nations (**FAO**) reports in 2023 that globally only 11% of all registered chicken breeds are not at risk, whereas 4% are vulnerable, 17% are endangered, 6% are critically endangered and 7% of the registered breeds are considered extinct (FAO, 2023). However, the remaining 56% of all registered chicken breeds has an unknown risk status. Therefore, it is clear that more research is necessary to genetically characterize the (local) chicken breeds of whom the risk status is unknown. Moreover, for those breeds that have been characterized, close monitoring is crucial (Meuwisen and Oldenbroek, 2017).

Several studies have already used genomic markers, such as single nucleotide polymorphisms (**SNPs**), to study and characterize (local) chicken breeds. For example, a study by Bortoluzzi et al. (2018) performed an extensive investigation on 41 chicken populations in the Netherlands, encompassing both commercial lines, (large fowl) fancy breeds and their corresponding bantam breeds. The authors could reveal the complete gene flow and genetic distinction between these populations and specifically between the large fowl breeds and their bantam counterparts. Additionally, the study explored the patterns of runs of homozygosity (**ROH**) in the different breeds. In 2019, Malomane et al. assembled a large dataset including 3,235 individuals from more than 160 international populations (called the *SYNBREED* panel/consortium) and performed several diversity analyses on this large set of breeds (including principle component analysis and phylogenetic trees). One of the interesting outcomes is that the African, South American, Asian, and European breeds harbored a remarkably large amount of genetic diversity. Recently, Restoux et al. (2022) performed a similar study including more than 25 local and commercial chicken breeds in France on which they performed ROH analyses, computed genetic distances and estimated effective population sizes. Moreover, they reported that breeds with consistent management, including pedigree recording, optimized mating plans and nonextreme selection, over a longer period of time showed less signs of recent inbreeding. However, they also detected the formation of within breed substructures according to the breeder's exchange practices, which could lead to genetic erosion of the breed. All these studies show the importance and interest of using whole genome SNP markers to study local breeds, especially when pedigree data are not available.

The emergence of SNP genotyping arrays has made it increasingly feasible to conduct whole genome marker studies, even for local breeds that are economically less prominent or smaller in size. However, for chicken there are only a few SNP arrays available at the moment. One is privately owned (approximately 60K markers) (Groenen et al., 2011) and was used in, for example, Bortoluzzi et al. (2018). Another is publicly available (around 580K markers) (Kranis et al., 2013) and was used for example in Malomane et al. (2019). Finally, Liu et al. (2019) recently announced a new Illumina-based array with 55K markers with a specific focus on Chinese local chicken breeds. However, it is important to emphasize that these arrays come with a relatively high per-animal cost, typically ranging between $100 and $175, and they have limited marker overlap among them, as reported in Tixier-Boichard et al. (2022). Therefore, the recently introduced IMAGE001 SNP genotyping array emerges as a valuable and cost-effective alternative for genotyping. This array is a multispecies chip for genomic assessment and includes 59,724 SNPs, divided over 6 livestock species (cow, pig, sheep, chicken, horse, and goat), resulting in approximately 10K SNPs per species. It was created within the *Innovative Management of Animal Genetic Resources* project (IMAGE; https://www.imageh2020.eu/) to aid in the characterization of gene banks and local livestock populations. The developers aimed to have at least 80% of all markers in overlap with other, already available arrays and approximately 20% of the markers are trait- or QTL-related, positioned on sex chromosomes or on the mitochondrional DNA. The IMAGE001array is available at a relatively affordable price (±20$/sample) and publicly available without restrictions (Tixier-Boichard et al., 2022). Also, the project consortium is developing a second array (IMAGE002) for 6 other species: water buffalo, duck, quail, bee, rabbit and pigeon. A first of test of the IMAGE001 array on more than 200 samples of more than 25 populations for chicken was presented in 2022 (Tixier-Boichard et al., 2022). The authors reported an average minor allele frequency (**MAF**) of 0.34, indicating that the tool is valuable for a large number of different populations. Moreover, they used this array successfully to perform principal component analyses (**PCA**) and neighbor-joining tree analyses to show that local breeds did not cluster based on geographical regions and that they were generally homogenous. Michiels et al. (2023) used the IMAGE001 array to characterize 3 cattle populations in Argentina, using PCA and *F*_ST_ analysis, where they found that the local Argentinean Creole cattle represents a unique genetic pool. They concluded that this cheap and accessible multispecies array could become a useful tool to monitor local breeds in both developed and developing countries. Nevertheless, the utilization of this array for characterizing chicken breeds remains unexplored at present.

In this study, our objective is to showcase the novel IMAGE001 array for the first time as a tool for investigating genetic diversity in a local small poultry breed. Consequently, this research focuses on characterizing a unique Belgian chicken breed: the Turkey-headed Malines (**THM**) (Mechelse Kalkoenkop/Coucou de Malines à tête de Dindon). This chicken breed is 1 of 38 local Belgian chicken breeds, and comprises of approximately 300 individuals, raised by more than 60 breeders mainly in Belgium (some in France and additionally Germany and the Netherlands) (Heylen and Martens, 2022). The breed was created as a broiler breed and as a heavier and taller version of the Malines chicken (**MC**). The MC breed is frequently described as a dual-purpose breed and is sometimes named as a slower-growing alternative to traditional broiler lines (e.g., Ross PM3) (Mueller et al., 2018). Historic records give no unambiguous conclusion as from which breeds THM originates, with the exception of the MC (Schollaert, 2015). Other breeds suggested in its creation include the Bruges Game fowl (**BG**), the Brahma, the Izegem Coocoo (**IC**), and the Flemish Coocoo (**FC**) (Schollaert, 2015). Breed management of the THM is performed by the Flemish breeding organization *Steunpunt Levend Erfgoed* (**SLE**) that focuses on local breeds in Flanders (Northern Belgium). Pedigree records are not routinely collected for the THM.

The research objectives of this work were 2-fold: i) to evaluate the use of the novel IMAGE001 multispecies SNP genotyping array for a local chicken breed that was never studied before and not included in the development of the array; and ii) to genetically identify the local THM chicken breed in comparison to 4 other presumed related local breeds as a first subset of local Belgian chicken populations to be genotyped using whole genome SNP array technology. Therefore, this research establishes the foundation for a comprehensive genetic assessment of the local Belgian chicken heritage, simultaneously showing the values and limitations of the novel IMAGE001 SNP genotyping array to the scientific community.

## MATERIALS AND METHODS

### Animal Genotyping

In total, 139 chicken of 5 different breeds were genotyped (Table 1) and care was taken to include animals from different THM breeders. Genomic DNA was extracted from blood cards (Gensaver 2.0, Ahlstrom, Finland), provided by *SLE*, using the Qiagen DNeasy Blood and Tissue Kit. These chicken were genotyped together with 24 sheep samples and 219 pig samples on 1 IMAGE001 genotyping array. Genotyping was performed by Eurofins AROS, Denmark. The IMAGE001 array contains 9,306 SNP variants for chicken (Tixier-Boichard et al., 2022).

**Table 1:** Information on the 5 chicken breeds included in this study, the number of samples per breed and the number of different breeders of whom animals were sampled.

Breed name	Local name	Abbreviation	Number of samples	Number of sampled breeders
Turkey headed Malines	Mechelse Kalkoenkop	THM	110	17
Malines Chicken	Mechels Hoen/Mechelse Koekoek	MC	16	13
Izegem Cuckoo	Izegemse Koekoek	IC	4	2
Flemish Cuckoo	Vlaamse Koekoek	FC	4	2
Bruges Game fowl	Brugse Vechter	BG	5	3

Genotype quality control was performed using PLINK 1.9 (Chang et al., 2015) following Anderson et al. (2010). Individual genotyping rate was checked (all >95%) and none of the samples had an outlying rate of heterozygosity (>3 SD). Possible parentage was verified using identity-by-state (**IBS**) similarity (PLINK's –*genome PI_HAT* measure). On SNP level, SNPs with unmapped genomic location (*n* = 78), on mitochondria (*n* = 90) or sex chromosomes (*n* = 135) and SNPs with a low call rate (<90%, *n* = 335) were not retained for the analysis. For ROH analysis, no minor allele frequency (**MAF**) or linkage disequilibrium pruning was performed following Meyermans et al. (2020). This quality controlled dataset contained 8,670 genotyped SNPs on all 139 chicken. The average MAF per population was calculated using PLINK's–*freq* function. For PCA and *F*_ST_ analyses, MAF pruning (>0.05, *n* = 1,113) and linkage disequilibrium (**LD**) pruning (*R*² < 0.75, *n* = 470) were performed and this resulted in a dataset with 7,087 genotyped SNPs on all 139 chicken.

### Diversity Assessment

Average SNP homozygosity per population was assessed using PLINK's –*het* function. The effective population size (*N*_e_) was estimated for THM based on LD following Waples (1991, 2006), and Weir and Hill (1980) via a custom R-script. For the 4 other populations (BG, IC, FC, and MC), *N*_e_ was not estimated due to too small sample size. ROH detection was performed using PLINK's scanning window approach (–*homozyg* function) with a minimal ROH length 1,000 kb. The number of minimal SNPs per ROH was calculated following Purfield et al. (2012), resulting in at least 20 SNPs. Minimal SNP density was set at 200 kb/SNP and gaps until 1,000 kb were allowed. Within a ROH, no heterozygote and only 1 missing SNP were allowed. Scanning window size was equal to 20 SNPs, and the threshold was set at 0.05. As the IMAGE001 array generates low-density genotypes, special attention was given to the genome coverage of the ROH analysis. This genome coverage was computed according to Meyermans et al. (2020) to get an accurate view of the screened proportion of the genome. Therefore the genomic inbreeding coefficient based on ROH (F_ROH_) was not computed based on the total *Gallus gallus* genome size, as reported in NCBI, but rather by dividing the total ROH length by the exact genome size that was screened in the analysis. The genome-wide overview of ROH patterns for THM was presented using the *qqman* package (Turner, 2018). F_ROH_ were computed both for the whole genome and for all macrochromosomes separately (GGA1–GGA10) (International Chicken Genome Sequencing Consortium, 2004). Population-wide ROH islands for THM were identified following Gorssen et al. (2021), with a detection threshold of *P* < 0.005 resulting in a minimal incidence of 54% in the population for each ROH island.

The 5 populations that were genotyped in this study were investigated using PCA via PLINK's –*pca* command. Results were plotted using R's *ggplot2* package (Wickham, 2016). Next, a neighbor net graph was created based on pairwise Weir and Cockerham's *F*_ST_ values (–*fst* in PLINK) and visualized using SPLITSTREE5 (Huson and Bryan, 2006). Finally, the overlap between the SNPs that passed quality control in this study was compared to the number of SNPs that were present on the other commonly used arrays for chicken SNP genotyping (Groenen et al., 2011; Kranis et al., 2013).

## RESULTS

Genotype quality with the novel IMAGE001 array was high, as all 139 genotyped chicken had a sufficiently high call rate (>95%). Also for the 24 and 221 sheep and pig samples that were genotyped on the same array, all samples had high genotyping call rates (>99%), showing good performance of the IMAGE001 genotyping array. The average MAF per population on this novel genotyping array is presented in Table 2 and was highest for BG (0.245) and lowest for IC (0.200). The average MAF for THM was estimated at 0.230. Across all 5 genotyped populations, average MAF was 0.246 (SD: 0.147). For both THM and MC, mean heterozygosity was 0.33 (SD = 0.045), whereas for IC, FC and BG estimated mean heterozygosity was somewhat higher (0.40, 0.38 and 0.38, respectively) although their sample sizes were very low. *N*_e_ for THM was estimated at 18 and when adjusted for sample size at 34. Estimates for the 4 other populations were not deemed reliable due to the low sample size.

**Table 2:** The average (and standard deviation between brackets) minor allele frequency (MAF) and the number of SNPs with MAF <0.05 for the 5 breeds studied in this study for the IMAGE001 genotyping array.

Breed	MAF	N _MAF < 0.05_
THM	0.230 (0.151)	1,406
MC	0.237 (0.155)	1,327
IC	0.200 (0.170)	2,652
FC	0.244 (0.168)	1,764
BG	0.245 (0.163)	1,539

For IC, FC, and BG it has to be noted that sample sizes were low (<=5). THM: Turkey-headed Malines; MC: Malines chicken; IC: Izegem Coocoo; FC: Flemish Coocoo; BG: Bruges Game fowl.

### Runs of Homozygosity

**Figure 1:**
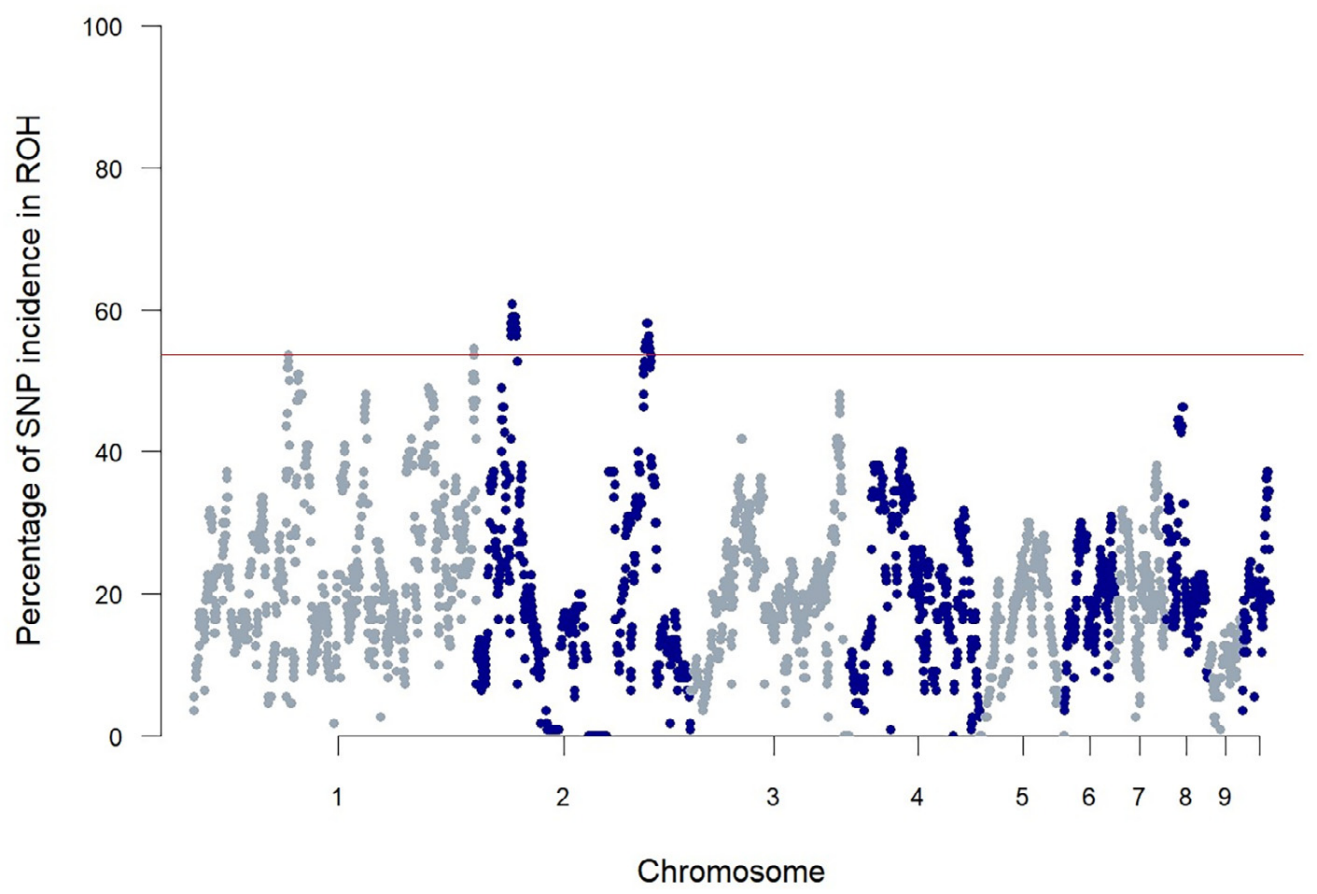
Overview of SNPs in ROH for the first 10 chromosomes (GGA1–GGA10) of THM chicken. Each dot represents the incidence of that SNP in a ROH (as percentage of the sampled population (*n* = 110)). The red line corresponds to a threshold of 54% incidence in the sampled THM population.

Table 3 shows the F_ROH_ calculated for all studied chicken populations. For THM, average F_ROH_ was highest, and 14 animals (13% of the sampled population) had an F_ROH_ > 0.33. The genome coverage of the ROH analysis was 83.8% of the whole chicken genome and 68.5% when analyzing only the first 10 chromosomes. When comparing the whole genome analysis vs. the analysis that only covers the first 10 chromosomes, Pearson correlation between F_ROH_ estimates is very high (>0.97). The ROH length of the detected ROH is also highly correlated (>0.98) and for example for THM, the difference between both methods (all chromosomes vs. only the 10 macrochromosomes) is on average 30.7 Mb (SD: 17.7) less. For THM chicken, the genome-wide overview of ROH patterns is shown in Figure 1 and shows several regions with increased occurrence of ROHs. Based on the 10-CHR analysis, 4 ROH islands were detected (Figure 1): on GGA1 around 66 Mb and around 195 Mb, and on GGA2 and around 24 Mb and around 118 Mb.

**Table 3:** Overview of the ROH analysis results for the 5 studied chicken breeds over the 10 macrochromosomes.

Breed	Average number of ROH	Total ROH length (kb)	F_ROH_	F_ROH > 5 Mb_	Max F_ROH_
THM	39.8	178.4	0.202 (0.097)	0.110 (0.072)	0.496
MC	42.8	173.9	0.198 (0.090)	0.094 (0.060)	0.345
IC	45.3	173.9	0.198 (0.031)	0.080 (0.015)	0.217
FC	40.5	186.2	0.212 (0.119)	0.103 (0.090)	0.360
BG	31.6	139.6	0.159 (0.085)	0.083 (0.057)	0.249

It has to be noted that for IC, FC and BG sample sizes were low (≤5). F_ROH > 5 Mb_ shows the F_ROH_ based on ROH fragments larger than 5 Mb. Max F_ROH_ shows the highest individual F_ROH_ in the sampled population. Standard deviations are given between brackets. THM: Turkey-headed Malines; MC: Malines chicken; IC: Izegem Coocoo; FC: Flemish Coocoo; BG: Bruges Game fowl.

### Interbreed Analysis

**Figure 2:**
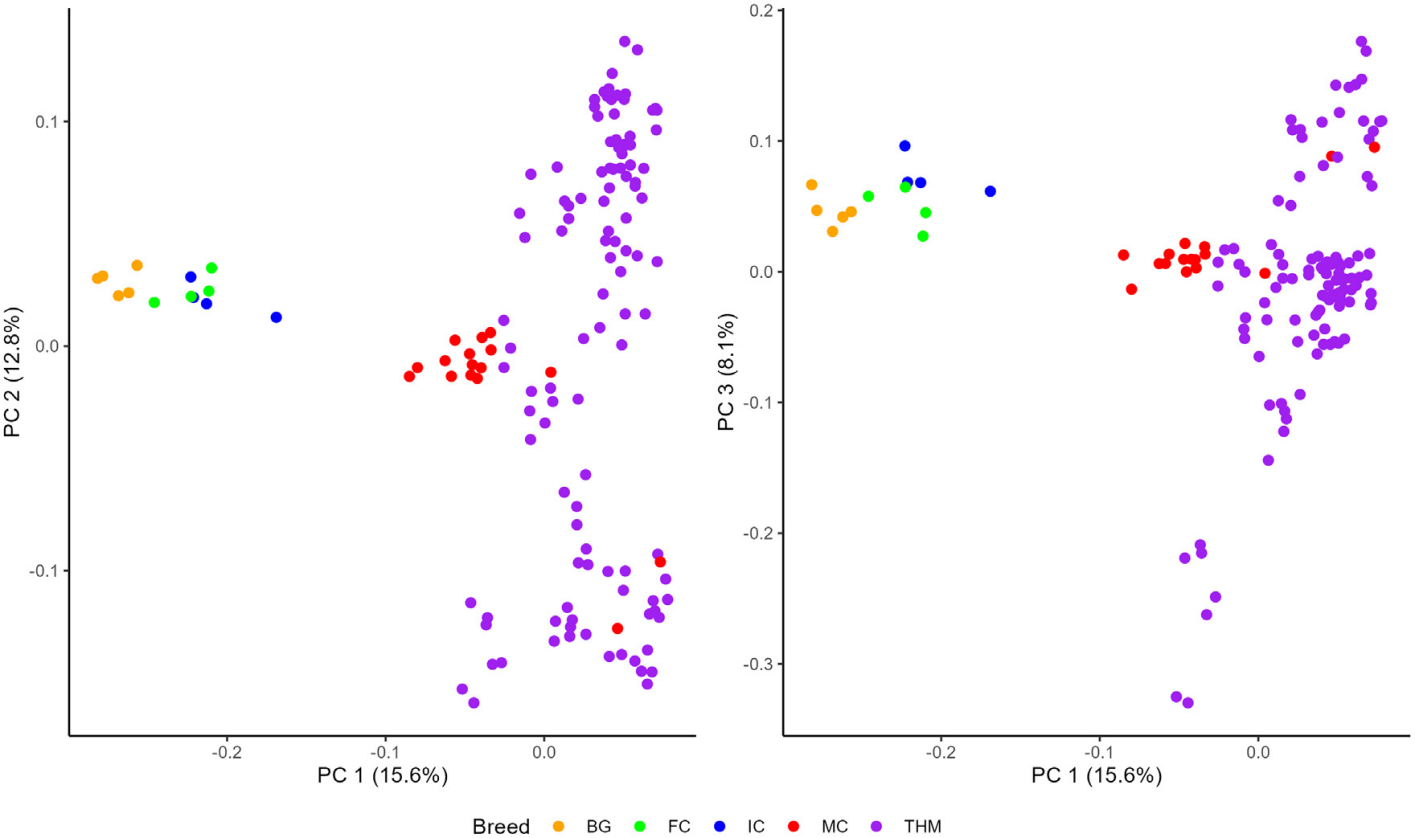
Visual plotting of the principal component analysis (PCA). The first principal component (PC) vs. the second PC (left) and the first PC vs. the third PC (right). The first PC explained 15.6% of the total observed variance, the second PC 12.8% and the third 8.1%. THM: Turkey-Headed Malines; MC: Malines chicken; IC: Izegem Coocoo; FC: Flemish Coocoo; BG: Bruges Game fowl.

Figure 2 shows the visual representation of the PCA for the first 3 principal components (**PC**) that explain a total of 36.5% of all observed variance. The first PC clearly discriminates the THM and MC breeds from the BG, FC and IC populations, whereas PC2 and PC3 mainly describe differences in variances within the THM breed. The MC group exhibits close clustering with the THM group, while the IC and FC populations cluster together, with the BG group appearing as the most distinct among the analyzed breeds. Two animals that are registered as MC cluster in the THM population. The first MC has a ± 0.60 IBS relationship with 2 other THM and is closely related (>0.25 IBS) to 5 other THM. The second MC has ±0.35 IBS relationship with 1 THM, and is owned by a breeder that also keeps THM chicken.

**Figure 3:**
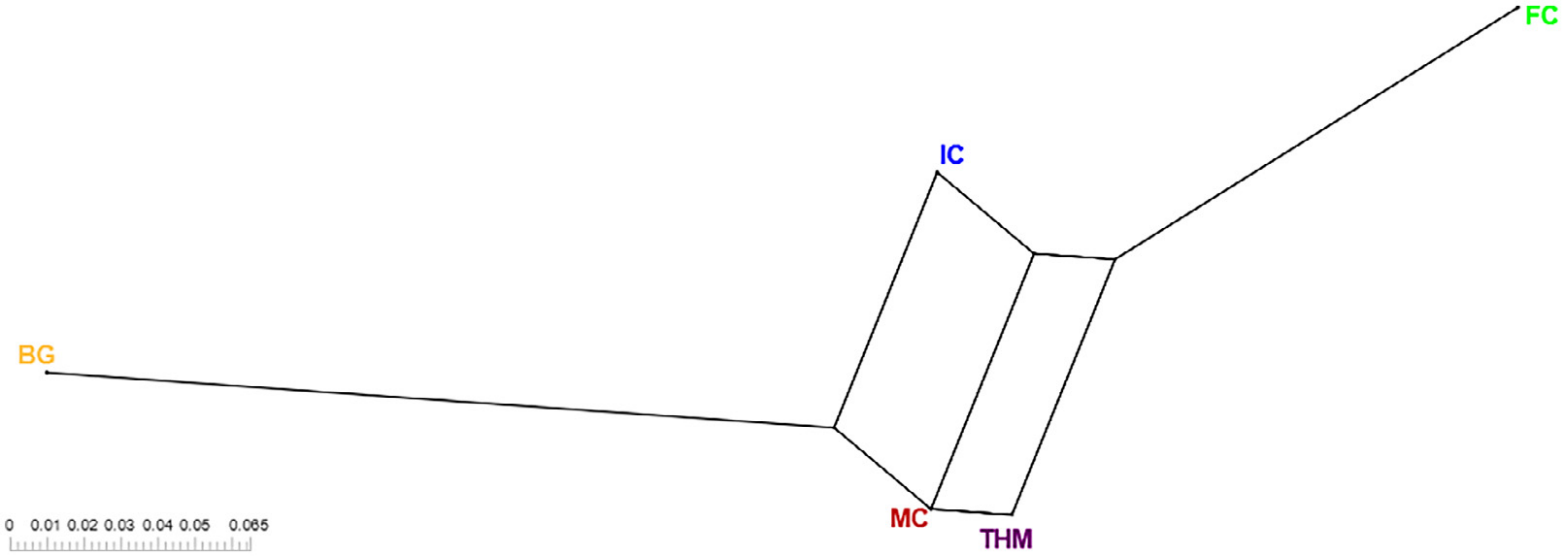
Neighbor net graph of the 5 populations genotyped in this study based on Weir and Cockerham's *F*_ST_ values. The scale legend is given on the left. THM: Turkey-Headed Malines; MC: Malines chicken; IC: Izegem Coocoo; FC: Flemish Coocoo; BG: Bruges Game fowl.

Based on *F*_ST_ analysis it is clear that the sampled THM and MC are closely related (*F*_ST_ = 0.042) whereas THM are clearly distinct from the 2 other Coocoo breeds (IC and FC; *F*_ST_ are 0.135 and 0.138, respectively). The neighbor net graph of the 5 breeds studied in this study is shown in Figure 3.

### IMAGE001 Array Compatibility

Finally, the SNP marker overlap between the IMAGE001 array and 2 other chicken genotyping arrays was investigated (Groenen et al., 2011; Kranis et al., 2013). Bortoluzzi et al. (2018) and Restoux et al. (2022) used a (custom) 60K Illumina Infinium iSelect array (Groenen et al., 2011) and retained 49,092, and 45,209 SNPs respectively after quality control. Of these, 4,310 SNP markers overlap with the 9,308 SNPs included on the IMAGE001 array (46%). Malomane et al. (2019) used the 580K Affymetrix Axiom Genome-Wide Chicken Genotyping Array (Kranis et al., 2013) of which 1,006 SNP markers overlapped with the 9,308 SNPs of the IMAGE001 array (11%).

## DISCUSSION

This study is the first to perform a whole genome diversity scan using the novel IMAGE001 genotyping to genetically characterize a local chicken breed array that was never studied before. In the present study, the THM, 1 of the local Belgian chicken breeds, was studied using ROH analysis, *N*_e_ estimation and compared to 4 other local chicken breeds (MC, BG, IC, and FC) using PCA and *F*_ST_ analysis.

### Performance of the IMAGE001 Array

The genotyping performance of the novel array was very high as all genotyped samples yielded good results. Mixing different species on the same array did not result in any problems and is an asset when sufficient samples need to be recruited to fill a 384-array. This might aid the periodic screening of gene bank collections. The average MAF of the genotyped SNP markers was somewhat lower than observed in Tixier-Boichard et al. (2022), which could be attributed to the relatively low genetic diversity in our examined populations. In Tixier-Boichard et al. (2022) a similar number of SNP markers passed QC (7,743 vs. 7,087 in the current study). In their study, they indicated that they would like to increase the number of markers passing QC in a second version of this array to reach the target of 10K SNPs for chicken. Overall, we deemed the IMAGE001 array successful for genetic characterization of our local chicken breeds and sufficient SNPs remained available for further analysis.

Moreover, we examined the overlap between the SNPs from the novel IMAGE001 array to the ones available via other medium density and 1 high-density genotyping array (Groenen et al., 2011; Kranis et al., 2013). Overall, overlap was intermediate (46%) to very low (11%). When merging these genotype datasets, only a maximum of 4K markers were found to be overlapping. We were not able to confirm that a PCA analysis on such merged dataset resulted in reliable results (results not shown). It is not certain that the most informative markers are exactly those that overlap between the different genotyping arrays. The relatively low overlap between IMAGE001 and other arrays was also reported in cattle, as Michiels et al. (2023) indicated an overlap of ±5K SNP markers between their genotyped Argentinian cattle and other online available, open access genotype populations. Therefore, this shows that the goal of at least 80% SNP overlap with other available genotyping arrays was not fully met. It is important to note that the main focus during development was likely not to maximize overlap with other arrays, as the primary goal was to design an array for diversity screening, which included important markers such as sex chromosomes, known traits, and MHC wherever possible. Additionally, it is worth noting that a second version of the array with an updated SNP panel is currently in progress (Tixier-Boichard et al., 2022).

### Diversity of THM

The LD-based *N*_e_ estimate for the THM breed is low (*N*_e_ = 34 when corrected for sample size), which is well below the general guideline by FAO (*N*_e_ ≥ 100) to maintain a sustainable long term population. This is a strong indication that the THM chicken breed is endangered. Restoux et al. (2022) computed pedigree-based *N*_e_ for 18 French chicken breeds and found estimates between 22 and 285 (median *N*_e_ = 89). Consequently, it can be concluded that the THM exhibits lower genetic diversity in comparison to the average French chicken breeds included in Restoux et al.'s (2022) research. Such low *N*_e_ calls for the setup of a strict conservation program for breeds, like the THM, with low to none commercial value. Conservation measures could include collection of pedigree data and promoting relatively underused sires for the next generation, but also the organization of exhibitions to raise awareness and support for the breed's preservation. Our research, along with international studies such as Restoux et al. (2022), underscores the vulnerability of local breeds and emphasizes the need for conservation efforts.

When studying inbreeding using ROH, it is clear that the average F_ROH_ for these Belgian chicken breeds is high, as also quite a number of individuals had a F_ROH_ > 0.33. Compared to the populations studied in Bortoluzzi et al. (2018) and Restoux et al. (2022), these F_ROH_ are very similar, as the average F_ROH_ for the 41 Dutch breeds in Bortoluzzi et al. (2018) averaged at 0.34 (range from 0.14 to 0.66) and for the 28 French breeds in Restoux et al. (2022) F_ROH_ averaged at 0.24 (range from 0.13 to 0.42). The average length of the estimated ROH was somewhat higher and the number of ROH per individual was somewhat lower in our work compared to these studies. This can be explained by the lower number of SNPs genotyped on the IMAGE001 array. When regarding F_ROH >5 Mb_, it is clear that a large proportion (±50%) of the observed inbreeding can be attributed to relatively large ROH segments. Following Curik et al. (2014), this could be attributed to inbreeding events that took place relatively recent, approximately up to 10 generations ago.

As the IMAGE001 array harbors only a limited number of SNP markers, it is very important to monitor genome coverage of the ROH analysis. The full genome length, as published by NCBI, is equal to 1.065 Gb (International Chicken Genome Sequencing Consortium, 2004), and was only covered for 84% by the ROH analysis. By calculating F_ROH_ following Meyermans et al. (2020), we automatically took this lower genome coverage into account. Moreover, we mainly investigated the first 10 (macro)chromosomes as these chromosomes are normal-sized (all >20 Mb) (International Chicken Genome Sequencing Consortium, 2004). Chicken have a large number of microchromosomes, with an average size of 6 Mb and some as short as 1 Mb or less. They contain approximately one third of the whole genome (McQueen et al., 1998). The IMAGE001 array has for some of these microchromosomes a very low number of SNP markers, making ROH analysis on these chromosomes impossible. Therefore, these microchromosomes were not included in this analysis. A genome coverage around 68% when analyzing the first 10 chromosomes shows that our analysis does cover almost the full size of the first 10 chromosomes.

Our ROH analysis revealed 4 different ROH islands in THM chicken (Figure 1). The first ROH island, located on GGA1 around 65.8 Mb is located near the *SOX5* (sex determining region Y-box 5) gene. This gene is known for its link to the pea comb phenotype in chicken. Wright et al. showed in 2009 that a copy number variant in this gene was linked to a reduced size in combs and wattles. Therefore, this region is somewhat overrepresented in SNP variants on the IMAGE001 array, which could lead to a overestimation of the number of true ROH in this region. In this case, 13 SNPs are present on the array within a 500 bp region near the *SOX5* locus. Nevertheless, the influence on of this 1 ROH on the overall F_ROH_ estimate is relatively low. The second ROH island on GGA1 at 195 Mb which is at the telomere of this chromosome, is close to more than 50 genes (located in the vicinity of the island (±1 Mb)). So the high number of detected ROH cannot simply be attributed to a mere ROH detection bias. For both signals on GGA2, no clear selection signature, that was previously described, could be identified. The detection of these ROH islands, such as on GGA1, highlights how selection for phenotypic traits, like the pea comb, can influences the genomic landscape of small breeds. This underscores the importance of maintaining a balance between selecting for specific traits and preserving genetic diversity.

### Interbreed Analysis

The PCA and F_ST_ analyses show the genetic differences between the 5 analyzed populations. Those differences correspond also to variations in morphological characteristics (Heylen and Martens, 2022). The THM was previously assumed to originate from MC, and closely related to the BG due to several admixture events. The close relationship of THM to MC was indeed observed (Figures 2 and 3) with an FST estimated at 0.042. However, the admixture of BG animals was not validated based on PCA analyses, as the BG clustered separate from THM/MC, IC and FC (Figure 3). Indeed, Heylen and Martens (2022) already pointed to the strongly hypothetical nature of the descendance of THM from BG. Genotyping more BG chicken could result in a better investigation of the putative admixture of BG into the THM.

The PCA analysis revealed 2 MC within the THM cluster. Further IBS examination pointed out that indeed both MC are highly related to several THM chicken, which could point either at a sample misidentification, or a false breed designation. As, MC and THM are highly similar morphologically, this cannot be excluded. More probable, however, is the mixing of THM with MC populations, as has happened sometimes during the last decades when breeders kept both chicken breeds. This shows another advantage of genotyping local livestock breeds: such “errors” cannot be picked up when breed management is only monitored by pedigree, if even available.

Although Lenstra et al. (2012) warn that unequal population sizes may cause bias in the outcome of PCA and admixture analyses, we deem this risk as low as we performed multiple PCA of subsets with maximum 15 THM and yielded highly comparable results (results not shown). Moreover, for *F*_ST_ analyses and the neighbor-joining tree (Figure 3) the chance on introducing bias is lower. Furthermore, special attention was given to the selection of the samples of MC, IC, BG and FC to select them as representative as possible for the whole population.

This study was the first to study the genetic diversity of the THM, a local chicken breed in Belgium. This was done using the novel multispecies SNP genotyping array IMAGE001. Genotyping performance of the array was high, and 8,670 SNPs were withheld for further analysis. ROH analysis revealed an average F_ROH_ of 0.202 and some highly inbred individuals. Effective population size estimation based on linkage disequilibrium revealed a low genetic diversity (*N*_e_ = 34), classifying the THM as endangered. PCA and *F*_ST_ analysis revealed the THM's relationship to other Belgian chicken breeds. A shortcoming of this novel array was the relatively low number of overlapping SNPs with other available SNP genotyping platforms. With this study, we demonstrate the effectiveness of affordable, low-density SNP genotyping arrays for assessing animal genetic resources and we established the basics for a thorough genetic evaluation of the Belgian local chicken breeds. Additionally, we would like to highlight the potential of the novel IMAGE001 genotyping array in characterizing other local breeds, contributing to broader efforts in the preservation and study of genetic diversity in poultry populations and gene banks worldwide.
